# Utilizing dynamic treatment information for MACE prediction of acute coronary syndrome

**DOI:** 10.1186/s12911-018-0730-7

**Published:** 2019-01-09

**Authors:** Huilong Duan, Zhoujian Sun, Wei Dong, Zhengxing Huang

**Affiliations:** 1 College of Biomedical Engineering and Instrument Science, Zhejiang University, Key Lab for Biomedical Engineering of Ministry of Education, Zheda Road, Hangzhou, China; 2 Department of Cardiology, Chinese PLA General Hospital, Beijing, China

**Keywords:** Acute coronary syndrome, MACE prediction, Deep learning, Bidirectional recurrent neural network, Electronic health record

## Abstract

**Background:**

Main adverse cardiac events (MACE) are essentially composite endpoints for assessing safety and efficacy of treatment processes of acute coronary syndrome (ACS) patients. Timely prediction of MACE is highly valuable for improving the effects of ACS treatments. Most existing tools are specific to predict MACE by mainly using static patient features and neglecting dynamic treatment information during learning.

**Methods:**

We address this challenge by developing a deep learning-based approach to utilize a large volume of heterogeneous electronic health record (EHR) for predicting MACE after ACS. Specifically, we obtain the deep representation of dynamic treatment features from EHR data, using the bidirectional recurrent neural network. And then, the extracted latent representation of treatment features can be utilized to predict whether a patient occurs MACE in his or her hospitalization.

**Results:**

We validate the effectiveness of our approach on a clinical dataset containing 2930 ACS patient samples with 232 static feature types and 2194 dynamic feature types. The performance of our best model for predicting MACE after ACS remains robust and reaches 0.713 and 0.764 in terms of AUC and Accuracy, respectively, and has over 11.9% (1.2%) and 1.9% (7.5%) performance gain of AUC (Accuracy) in comparison with both logistic regression and a boosted resampling model presented in our previous work, respectively. The results are statistically significant.

**Conclusions:**

We hypothesize that our proposed model adapted to leverage dynamic treatment information in EHR data appears to boost the performance of MACE prediction for ACS, and can readily meet the demand clinical prediction of other diseases, from a large volume of EHR in an open-ended fashion.

## Background

Acute coronary syndrome (ACS) is a term used to describe a range of conditions associated with sudden, reduced blood flow to the heart, including ST-elevation myocardial infarction (STEMI), non- ST-elevation myocardial infarction (NSTEMI), and unstable angina (UA) [1]. ACS is the most common type of coronary artery disease (CVD) [2–4]. Every year, CVD and ACS together account for approximately 7 million deaths [5, 6], accounting for around half of the global burden [7], and about 30% people are at risk of having ACS during their lifetime [8].

Main adverse cardiac event (MACE) refers to a type of composite end-event point event that contains unstable angina, myocardial infarction, death, and revascularization during hospitalization. As a vital composite endpoint, MACE has been frequently used in assessing safety and efficacy of treatment processes of ACS patients [9–12]. MACE prediction can be used to anticipate whether an individual is likely to experience unexpected adverse cardiac events during his or her hospitalization and after discharge [5, 13, 14]. Traditionally, cohort-based studies are conducted to develop MACE prediction tools. Recently, with the increasing availability of a large volume of electronic health record (EHR) data, there is a gradual attention to use data-driven approaches to construct efficient tools for MACE prediction [12, 15–17]. Theoretically speaking, the two types of studies have different concerns. Cohort-based studies are usually based on a small set of handpicked patient features which are collected in costly trials, and the generated tools are relatively simple to use in clinical practice. On the contrary, EHR data-driven models can remedy the limitations of cohort-based studies, but are usually complex and difficult to interpret. Although valuable, most existing models proposed by both types of studies have a common serious limitation, i.e., they are built on static patient features and neglect the influence of dynamic treatment information on MACE prediction. Since there have been considerable evidence that clinical conditions of patients are dynamically changed when treatments are performed, dynamic treatment information has the potential to boost the performance of MACE prediction.

To this end, this study proposes a data-driven model that leverages recurrent neural networks (RNN) to learn the deep feature representation of dynamic features which are extracted from EHR [18–20]. In comparison with traditional temporal analysis methods, such as the Cox proportional hazard model [21], RNN provides a substantial nonlinear improvement in model generalization and is more scalable [7]. RNN has proven effective in many difficult machine learning tasks, such as image processing [18] and language translation [19]. To fully utilize the dynamic information and avoid the influence of gradient vanishing, we adopt a specific type of RNN structure, i.e., bi-directional RNN with long-short time memory (Bi-LSTM) [20, 22], to extract dynamic features from EHR data to predict MACE. To our best knowledge, this is the first work for MACE prediction by taking into account not only the static patient features but also dynamic treatment information into a deep neural network model.

The contributions of this paper can be summarized as follows:

- We present a deep learning model to utilize dynamic treatment information for predicting MACE after ACS, and the incorporation of dynamic treatment information into learning boosts the performance of MACE prediction.
- The proposed model extracts the latent representation of dynamic treatment features via Bi-LSTM, which can be used to predict whether a patient occurs MACE in his or her hospitalization.
- Extensive experiments are conducted on a real EHR dataset, which consists of 2930 ACS patient samples collected from a Chinese hospital, to demonstrate the effectiveness of our proposed model for MACE prediction.

The remainder of this paper is organized as follows. The related work is introduced in Section 2. In Section 3, we present our proposed model of utilizing dynamic treatment information for predicting MACE after ACS, via a typical deep neural network architecture, i.e., Bi-LSTM. In Section 4, we present experimental results and evaluate the performance of our method in comparison with the state-of-the-art models. The merits and limitations of our proposed model are discussed in Section 5. Finally, Section 6 concludes our work and discusses future directions.

## Related work

From the technique perspective, the work on MACE prediction can be categorized as cohort-based studies and data-driven studies, respectively.

As a traditional approach of medical research, cohort-based studies have been widely adopted to investigate specific clinical hypothesis questions, e.g., the relationship between potential risk factors and MACE [23, 24]. In general, a hypothetical question is firstly proposed by clinical researchers, and then a group of subjects are recruited into the cohort and observed over a period, to collect data that may be relevant to the hypothesis. The prediction models can be furtherly developed based on the collected cohort data, via univariate, multivariate logistic regression or Cox proportional hazards regression model, etc. The most famous cohort-based models for MACE prediction include the Global Registry of Acute Coronary Events (GRACE) [2], the Thrombolysis in Myocardial Infarction (TIMI) [3], and the Platelet Glycoprotein IIb/IIIa Unstable Angina: Receptor Suppression Using Integrilin Therapy (PURSUIT) [5], etc.

Although useful, there is a serious flaw of cohort-based studies: they usually select a small set of patient variables, to simplify the model and facility its use in clinical practice [25]. However, the inclusion of fewer risk factors into the model learning may lead to the degradation of the model’s predictive performance. On the contrary, more potential risk factors (e.g., Cystain C, homocysteine in MACE prediction) are recently identified in the literature [26], but are not included in the existing cohort-based models, and therefore eventually limits the value of the cohort-based models.

Recently, with the widely application of EHR in healthcare facilities, thousands of data-driven models have been developed by exploring the huge potential of EHR data in various clinical applications, e.g., screening, diagnosis, treatment, prognosis and monitoring [27]. Compared with the traditional cohort-based studies, EHR data-driven models can well address the limitations of cohort-based studies [25].

Early work on data-driven prediction has been performed based on conventional machine learning and data mining methods. For example, Hu et al. proposed a hybrid model that combines both random forest and support vector machine to predict the risk of MACE [11]. Bandyopadhyay et al. proposed a Bayesian network to predict cardiovascular risk [28]. In [29], a vector spline multinomial logistic regression model was presented to predict risks of patients with ovarian tumors. These works show the usefulness of utilizing medical data for clinical risk prediction. Recently, many deep learning models, e.g., Stacked Denoising Auto-encoder (SDAE), and Convolutional Neural Network (CNN), etc., have been adopted for the prediction/detection task in medical domain and achieved a promising performance. For example, Raghavendra et al. proposed a CNN-based model to diagnosis the glaucoma using digital fundus images [15], and afterwards they applied CNN to detect the myocardial infarction and ventricular arrhythmias in ECG singles [16, 17]. Huang et al. proposed a regularized SDAE to predict the risk of ACS patients [30]. Li et al. developed a deep belief network based model to predict the risk factors of bone disease progression [31].

Although successful, not the full potential of EHR data has been explored. To the best of our knowledge, most of existing data-driven models were trained based on static patient features, and lack the ability to model time-dependent co-variates in the observation window such that an individual’s disease progression mediated by dynamic treatment information cannot be reliably measured, which limits the performance of predictive models.

## Methods

**Fig. 1:**
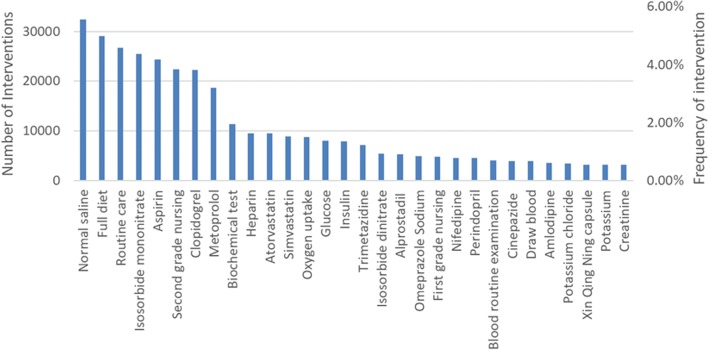
Utilizing EHR data to support MACE prediction during ACS patients’ hospitalization

Figure 1 illustrates our idea of utilizing temporal treatment information for MACE prediction of ACS patients during their hospitalizations. More details of our approach are presented as follows:

### Patient feature processing and embedding

**Fig. 2:**
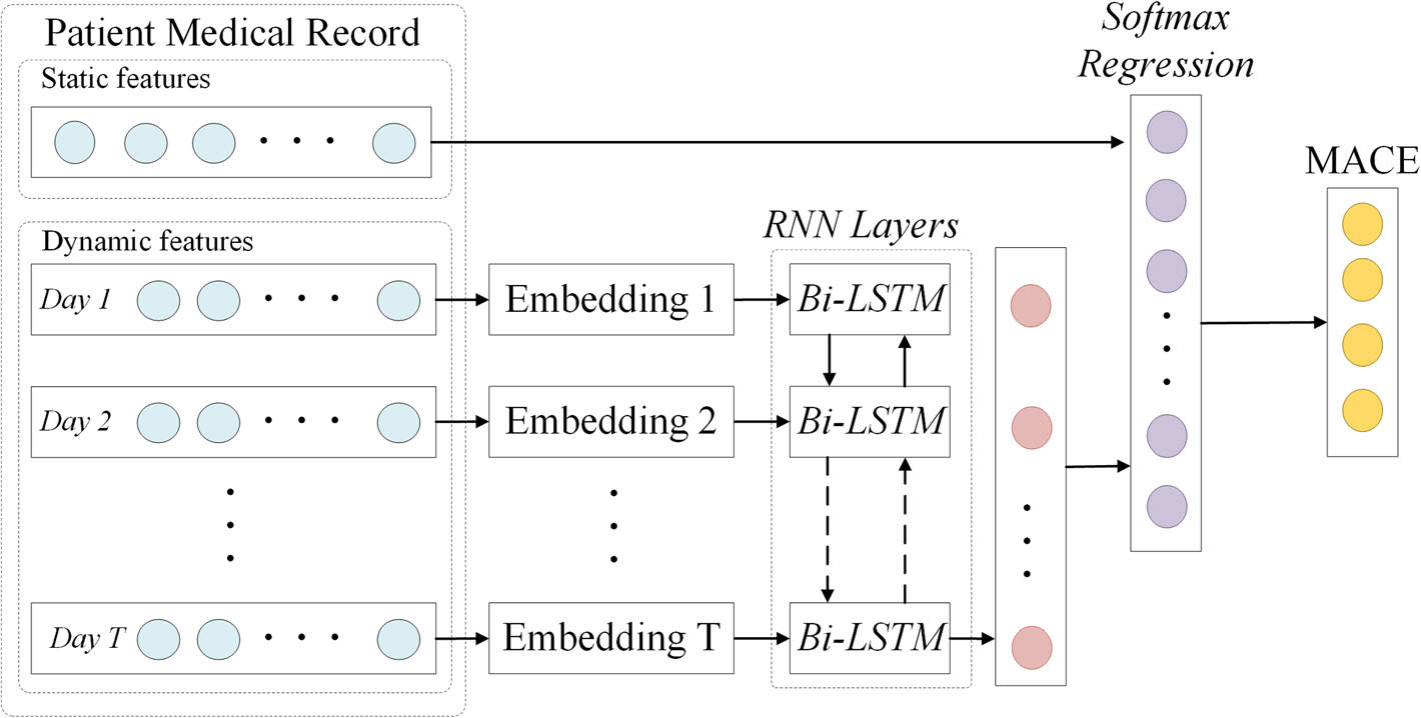
Representing dynamic features found on each hospitalization day as a specific vector

Clinical information is regularly observed/recorded in EHR data. From the temporal perspective, clinical data of a patient’s EHR ***d*** = 〈***x***_s_, ***X***_d_〉 can be categorized as both static features ***x***_s_ and dynamic features ***X***_d_. As shown in Fig. 2, our encoders firstly map the dynamic part of an input to a sequence of *K*-dimensional embeddings ***X***_d_ ***=*** (***x***^(**1**)^, ***x***^(**2**)^,  … , ***x***^(*T*)^), using a lookup table with one vector for each time epoch (e.g., one hospitalization day in this study), where ***x***^(*t*)^ ***=*** [*x*_1_, *x*_2_⋯, *x*_*K*_] (1 ≤ *t* ≤ *T*) is the encoded vector, *K* is the cardinal number of dynamic features, and *T* is the length of stay (LOS) of that patient. The dimensions of dynamic features observed in that time epoch are set to 1 and the rest are 0.

The aim of this study is to utilize dynamic treatment information to boost the performance of MACE prediction for ACS patients. To this end, we need to deal with information that may cause MACE, i.e., the record happened before MACE. To this end, we need to truncate our data to eliminate the influence of clinical information that would not cause MACE. Specifically, if an adverse cardiac event is observed at time stamp *t* for a particular patient sample 〈***x***_s_, (***x***^(**1**)^, ***x***^(**2**)^,  … , ***x***^(*t*)^,  … , ***x***^(*T*)^)〉 with LOS *T*, the prefix of the original input sequence, i.e., 〈***x***_s_, (***x***^(**1**)^, ***x***^(**2**)^,  … , ***x***^(*t*)^)〉, is selected as a training sample (〈***x***_s_, (***x***^(**1**)^, ***x***^(**2**)^,  … , ***x***^(*t*)^)〉, *c*), where *c* ∈ {0 : none, 1 : MACE } denotes the MACE label at time stamp *t*. If multiple MACEs are observed in one patient’s hospitalization, the firstly happened MACE and its’ previous observed treatment information are selected to be the MACE label and the training sample, respectively.

Note that, (***x***^(**1**)^, ***x***^(**2**)^,  … , ***x***^(*T*)^) is a sequence of dynamic features observed during a patient’s hospitalization. Intuitively, we initialize the representation for these dynamic features of each individual patient. A widely adopted strategy is to represent each word (a.k.a. dynamic feature) by using one-hot vector. However, these dynamic features observed on a specific time-period (i.e., one day, etc.) and thus may have no strict order. In fact, these dynamic features are treatment interventions, which can be performed on the patient in a loosely-structure manner [32]. Therefore, as depicted in Fig. 2, we embed the set of dynamic features observed on each hospitalization day to a vector, and then apply the standard Bi-LSTM to encode contextual semantic representations for dynamic features.

### Using Bi-LSTM to generate deep representations of treatment information

**Fig. 3:**
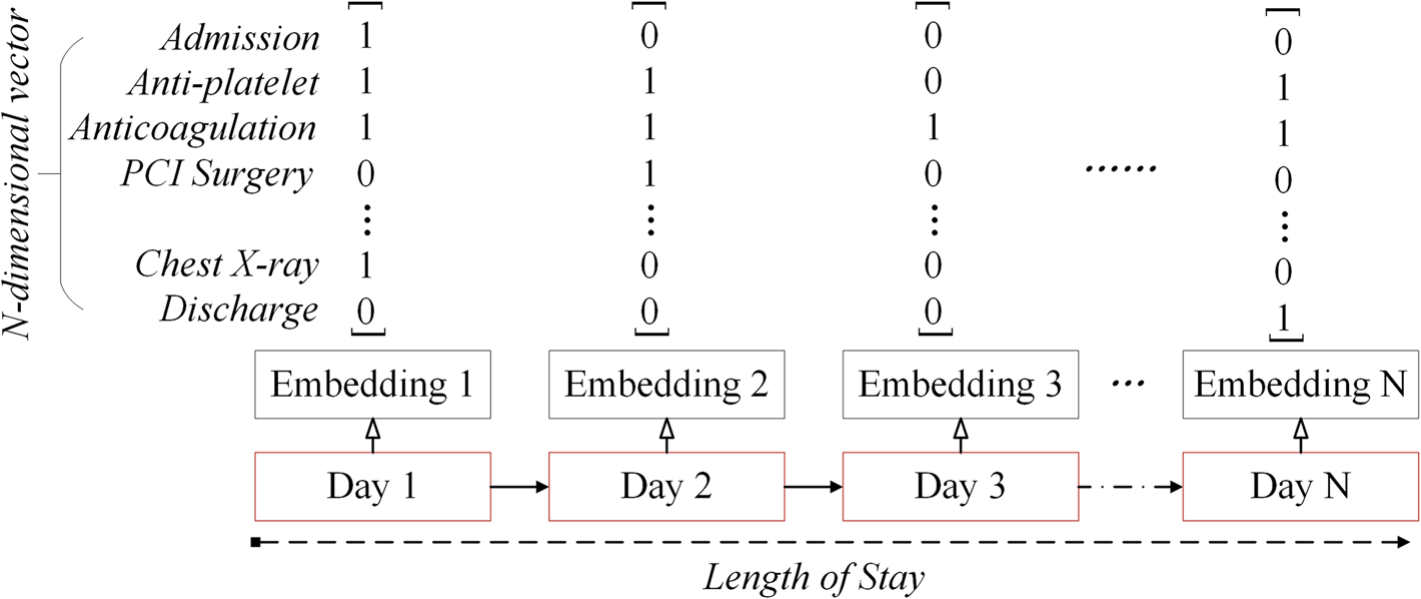
The framework of bi-directional LSTM used in this proposed MACE prediction model

Our method assumes use of both RNN and logistic regression to predict MACEs of ACS patients during their hospitalizations. The simplified layer architecture that generates deep representation of our mix model is presented in Fig. 3.

Original RNN is a neural network architecture designed to handle sequential input data, but it lacks the ability to model long-term dependencies. A LSTM is a type of RNN cell that addresses this issue by keeping a memory cell to serve as a summary of the preceding elements of an input sequence. In this study, we adopt Bi-LSTM to extract deep features from dynamic treatment information in EHR data. As can be seen in Fig. 3, it has four kinds of layers: input layer, dynamic feature-embedding layer, forward hidden layer, and backward hidden layer. The input layer encodes temporal treatment information extracted from raw EHR data. Then the dynamic feature-embedding layer converts the treatment information to an embedding vector, whose details are explained above. After dynamic feature-embedding layer, there are two parallel LSTM layers: forward hidden layer and backward hidden layer. At each time-step *t*, the forward hidden layer will compute a hidden representation $\left({\overrightarrow{\boldsymbol{h}}}_1,\cdots, {\overrightarrow{\boldsymbol{h}}}_t\right)$ of the sub-sequence that contains treatment information from ***x***_1_ to ***x***_*t*_. For the backward LSTM, it processes each treatment sequence in its reverse order, and forms a sequence of hidden representation $\left({\overleftarrow{\boldsymbol{h}}}_1,\cdots, {\overleftarrow{\boldsymbol{h}}}_t\right)$ of the sub-sentence that contains treatment information from ***x***_*t*_ to ***x***_1_. We calculate the hidden states ${\overrightarrow{\boldsymbol{h}}}_t$ by the following equations:

1 $$ {\boldsymbol{i}}_t=\sigma \left({\boldsymbol{W}}_i{\boldsymbol{x}}_t+{\boldsymbol{U}}_i{\overrightarrow{\boldsymbol{h}}}_{t-1}+{\boldsymbol{b}}_i\right) $$

2 $$ {\widehat{\boldsymbol{C}}}_t=\tanh \left({\boldsymbol{W}}_c{\boldsymbol{x}}_t+{\boldsymbol{U}}_c{\overrightarrow{\boldsymbol{h}}}_{t-1}+{\boldsymbol{b}}_c\right) $$

3 $$ {\boldsymbol{f}}_t=\sigma \left({\boldsymbol{W}}_f{\boldsymbol{x}}_t+{\boldsymbol{U}}_f{\overrightarrow{\boldsymbol{h}}}_{t-1}+{\boldsymbol{b}}_f\right) $$

4 $$ {\boldsymbol{C}}_t={\boldsymbol{i}}_t\cdotp {\widehat{\boldsymbol{C}}}_t+{\boldsymbol{f}}_t\cdotp {\boldsymbol{C}}_{t-1} $$

5 $$ {\boldsymbol{o}}_t=\sigma \left({\boldsymbol{W}}_o{\boldsymbol{x}}_t+{\boldsymbol{U}}_o{\overrightarrow{\boldsymbol{h}}}_{t-1}+{\boldsymbol{b}}_o\right) $$

6 $$ {\overrightarrow{\boldsymbol{h}}}_t={\boldsymbol{o}}_t\cdotp \tanh \left({\boldsymbol{C}}_t\right) $$

where *σ* represents the sigmoid activation function, *W*_∗_ is the input-to-hidden weight matrix, *U*_∗_ is the state-to-state recurrent weight matrix, and *b*_∗_ is the bias vector. The hidden state of LSTM is the concatenation of $\left({\boldsymbol{c}}_t,{\overrightarrow{\boldsymbol{h}}}_t\right)$. The long-term memory is saved in ***c***_*t*_, and the forget gate and input gate are used to control the updating of ***C***_*t*_, and the output gate is used to control the updating of ${\overrightarrow{\boldsymbol{h}}}_t$.

To make full use of information hidden in Bi-LSTM, we merge the hidden representations of forward and backward layers by concatenating ${\overrightarrow{\boldsymbol{h}}}_{\mathrm{t}}$ and ${\overleftarrow{\boldsymbol{h}}}_1$., i.e., the last states of both layers, Hence, the output representation of Bi-LSTM layer can be denoted as ${\boldsymbol{h}}_{\mathrm{e}}=\left[{\overleftarrow{\boldsymbol{h}}}_{\mathrm{t}},{\overrightarrow{\boldsymbol{h}}}_1\right]$. The output hidden layer ***h***_e_ of input sequence ***x***_e_ is then incorporated with static patient features ***x***_s_, which is already compressed, to represent the final state of the patient sample ***z*** = [***x***_s_, ***h***_e_].

### MACE prediction

To predict a distribution *P*(*y*_*i*_) over MACE outcome *y*_*i*_ ∈ *C*, the outputs ***z***^(*i*)^ are passed through a logistic regression layer ${\widehat{y}}_{\mathrm{i}}=\upsigma \left({\mathbf{W}}_{\mathrm{z}}{\boldsymbol{z}}^{(i)}+{b}_z\right)$, where **W**_z_ and *b*_*z*_ are learned parameters for logistic regression.

To learn the parameters of the proposed model, we set the cross-entropy of *y* as the loss function and minimize it in terms of ***W***_*r*_***,W***_*c*_***,W***_*f*_***,W***_*o*_***,W***_*z*_ and *b*_*z*_. As our model is a supervised method, each patient sample ***x***^(*i*)^ has its golden MACE outcome *y*^(*i*)^. The following loss function is used:

7 $$ \mathrm{Loss}=-\frac{1}{\left|D\right|}{\sum}_{i=1}^{\left|D\right|}\left({c}^{(i)}\mathit{\log}{y}^{(i)}+\left(1-{c}^{(i)}\right)\log \left(1-{y}^{(i)}\right)\right) $$

where ∣*D*∣ is the total number of training samples, *c*^(*i*)^ is the MACE indicator for the *i*-th patient where 1 indicates the occurrence of MACE and 0 control, and *y*^(*i*)^ is the output of the proposed model for an input ***x***^(*i*)^. The weights are updated during the training phase. Dynamic feature embeddings are fine-tuned as well. Optimization is performed using the back-propagation and the mini-batch stochastic gradient descent strategy.

## Results

### Data collection

This is a retrospective study assessing the performance of MACE prediction. The experimental dataset was extracted from the cardiology department of Chinese PLA General Hospital. Patients with ACS who admitted to the hospital between 2012 and 2016 were randomly selected in the experimental dataset. Three experienced clinicians were employed to ascertain MACE by reviewing medical records with a majority voting strategy. The experimental EHR dataset contains data for 2930 ACS patients. The dataset documented 233 static patient features including demographics, smoking status, alcohol consumption, laboratory values and diagnosis captured at the admission stage, etc. The dynamic data, including temporal treatment and care activities delivered during patients’ hospitalization, with 2194 features, is documented in EHR as well as static ones.

To be more specific, static patient features including demographic variables (e.g., age and gender), physical examination variables (e.g., blood pressure, heart rate and BMI), comorbidities, laboratory results, and disease/treatment history (e.g., Post-PCI, Post-CABG) were collected at the admission stage. All static features are time-invariant in a single hospitalization visit. Comorbidities were categorized as present or absent at the admission stage of patients’ hospitalizations. Variables with more than 30% missing values were not included in the analysis while the missing data belong to variables with less than 30% missing values is set by the median of that variable. Table 1 shows details of some critical static patient features.

**Table 1:** Baseline characteristics of the experimental dataset

Characteristics	No. of participants (*n* = 2930)
Demographics	
Age, (mean ± sdv.) [min-max]	62.27 ± 12.11 [28–91]
Gender, Male/Female	2079/851
Physical examination, (mean ± sdv.) [min-max]	
Systolic BP, mm Hg	132.10 ± 17.64 [11–240]
Diastolic BP, mm Hg	77.59 ± 10.17 [35–120]
Height, cm	167.01 ± 8.15 [56–188]
Weight, kg	71.81 ± 12.42 [32–200]
Ejection Fraction, (mean ± sdv.) [min-max]	59.51 ± 7.82 [17–78]
Comorbid conditions (%)	
Diabetes	803 (27.4%)
Hypertension	1981 (67.6%)
Heart Failure	165 (5.6%)
arteriosclerosis	2267 (77.4%)
History of current or previous smoking	1113 (38.0%)
Laboratory data, (mean ± sdv.) [min-max]	
Creatinine, umol/L	78.72 ± 38.18 [29.5–739.4]
Creatinine kinase, umol/L	86.11 ± 112.02 [6.2–4651.1]
Alanine aminotransferase, umol/L	26.02 ± 27.66 [1.7–593]
Aspartate aminotransferase, umol/L	21.70 ± 19.53 [5.8–589.4]
Troponin T, ng/ml	0.029 ± 0.084 [0.002–0.886]
Glucose, umol/L	6.16 ± 2.26 [2.69–28.62]
Disease/Treatment history (%)	
Post-PCI (patient who has taken PCI surgery in the past and was admitted into the hospital at this time)	816 (27.8%)
Post-CABG (patient who has taken CABG surgery in the past and was admitted into the hospital at this time)	46 (1.6%)
Length of Stay, (mean ± sdv.) [min-max]	9.12 ± 7.05 [1–54]
MACE (%)	752 (22.4%)

**Fig. 4:**
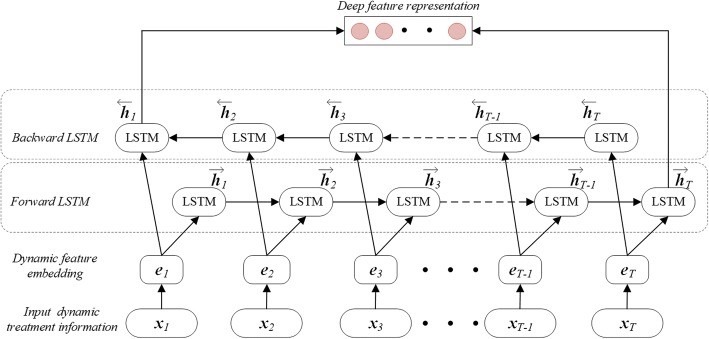
Number and frequency of top 30 treatment interventions in the collected EHR dataset

Dynamic features refer to medical interventions and their occurring time-stamps. Figure 4 plots both names and occurring time-stamps of top 30 most used treatment interventions contained in the experimental dataset. These 30 interventions occupy almost 50% treatment behaviors for ACS patients, while the other 2194 interventions occupy the left 50%, indicating that they are infrequently adopted in ACS patients’ treatment processes such that our dataset is very sparse.

Approval by the Data Protection Committee at the Chinese PLA General Hospital was obtained prior to initiation of the study. Informed consent was waived because of the presence of de-identified data and lack of feasibility of obtaining informed consent from all participants in the experimental dataset. A local ethics committee ruled that no formal ethics approval was required in this particular case.

### Experiment settings and baseline models

We present the following four learning strategies for MACE prediction:

1. Logistic regression based on static patient features, named LR. We build a LR classifier solely based on static patient features to construct a MACE prediction model. This model does not use dynamic treatment information into learning.
2. A boosted resampling model presented in our previous work [12], named Boosted-RMTM. This model was built based on static patient feature and dynamic treatment information was not used during learning, neither.
3. The proposed model was built based on both static patient features and dynamic treatment information, named mix model.
4. The proposed model was built based on dynamic treatment information, named dynamic model. This model does not use static patient features into learning.

We employ the four models to verify that if the utilization of dynamic treatment information can boost the performance of MACE prediction for ACS patients. The accuracy and AUC are selected as evaluation metrics in the experiments. These measure criterions are widely used in the evaluation of classification and prediction tasks in clinical applications.

All four models were trained using the experimental dataset. We iteratively divided the data into the train and test set with a ratio of 4:1, and reported the model performance on the test set. Specifically, we took four folds of data as the training set and the remaining one-fold as the test set. We conducted this for five different folds and calculated the average performance. Both the dynamic and mix models were trained ten times because of the non-convex character of the neural network. The results obtained by both dynamic and mix model are the average performances of 10 results. The performance of both Boosted-RMTM and LR was derived from our previous work [12].

As to the hyper-parameter setting of the proposed models, the learning rate is 0.01, the L2 coefficient is 0.01, the epoch is 200, the hidden state of Bi-LSTM is 256, and the static data is transformed into a 128-dimensional vector. To avoid overfitting, we used early stop and weight decay as regularization tricks. The proposed models were implemented by Python 3.5 in the Tensor flow framework. The source code is available in https://github.com/ZJU-BMI/mace_prediction.

### Data analysis

In the experiments, we used two metrics, i.e., AUC and accuracy, to evaluate the performance of MACE prediction. The value of AUC is invariant to the calibration [33]. Regarding the accuracy, different models have their specific optimal thresholds, which derives different calibration strategies [34]. However, it would be inappropriate to compare the performance between models if these models are calibrated with different strategies. To this end, we selected 0.5, which is the widely used threshold in literature, in our experiments for measuring the performance of MACE prediction.

Table 2 shows the evaluation results on both accuracy and AUC. Specifically, the proposed dynamic model obtains the best result in terms of both AUC and accuracy for MACE prediction. It indicates that the utilization of dynamic treatment information can improve the prediction performance for ACS. As can be seen in Table 2, the proposed mix model does not achieve expected performance for MACE prediction. The performance of the mix model is even worse than that of Boosted-RMTM. It is possible that the static data is sparser than that of dynamic data, and thus deteriorates the performance for MACE prediction.

**Table 2:** Experimental results with 7 days’ dynamic information

	AUC	Accuracy
LR	0.637 ± 0.010	0.752 ± 0.007
Mix	0.681 ± 0.006	0.746 ± 0.005
Dynamic	**0.713 ± 0.005**	**0.764 ± 0.004**
Boosted-RMTM	0.700 ± 0.003	0.689 ± 0.004

Note that there are just 22.5% patient samples who have MACE during their length of stay (as shown in Table 1), indicating that our dataset is typically imbalanced. To enhance the generalize ability of our model, some sampling tricks, such as over-sampling or under-sampling, can be imposed to address the data imbalance problem. We are sure that incorporating the proposed RNN-based learning strategy into the boosted resampling framework can further improve the performance of MACE prediction. However, it is beyond the scope of this study, and we plan to implement it in our future work.

#### Effect of length of stay

The longer one patient stays in a hospital, the more EHR data are accumulated. Intuitively, we hold the idea that the learning model will perform better if more data are integrated into learning. To validate this assumption, we explore the tendency of performance with the different length of stay.

**Fig. 5:**
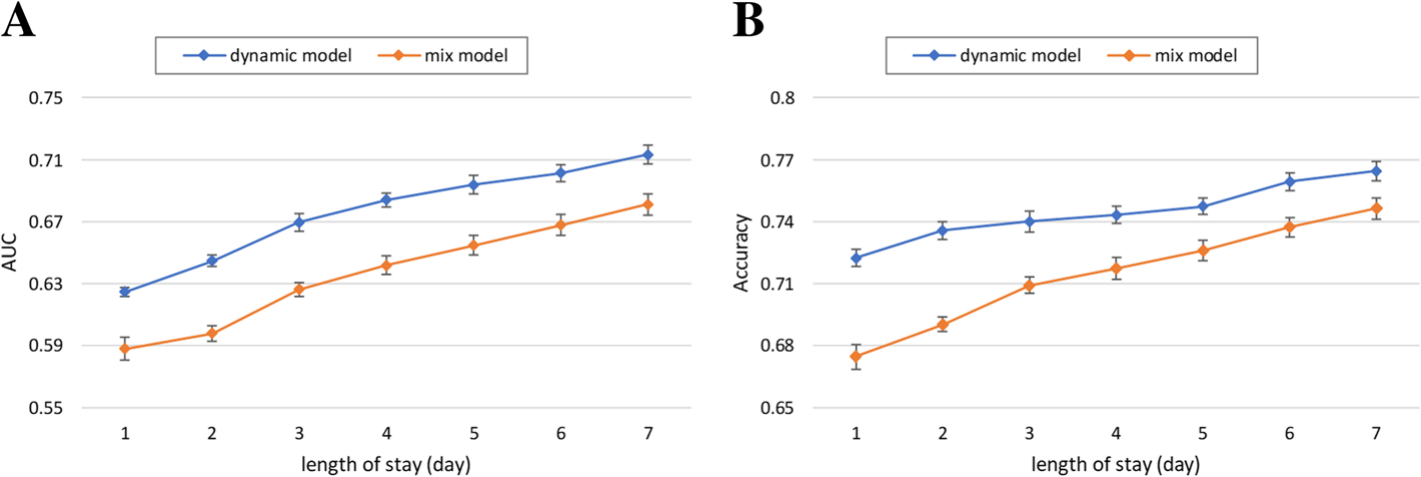
Impact of the different length of stay on the performance of MACE prediction regarding (**a**) AUC and (**b**) Accuracy

As can be seen in Fig. 5, we notice that both the AUC and accuracy curves dramatically increases with the increase of LOS. It indicates that incorporating more dynamic treatment information into learning can boost the prediction performance. Also, we can see from Fig. 5, that the dynamic model significantly performs better than the mix model regardless of the increase in LOS. This phenomenon, which is similar to the result listed in Table 2, proves the static features indeed deteriorate the performance.

#### Effect of training set size

**Fig. 6:**
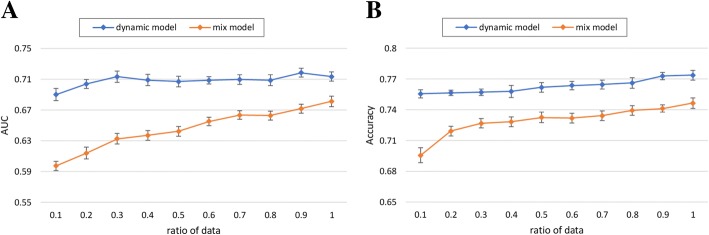
Impact of the different ratio of training data for MACE prediction, in terms of (**a**) AUC, and (**b**) Accuracy

Next, we study how the proposed approach performs with the increasing size of experimental data. As presented in Fig. 6a, the proposed dynamic model converges using only about 30% of data in terms of AUC, while the curve of the mix model increases consistently with the increase in the ratio of size. Figure 6b shows similar trends in terms of accuracy. The curve of the dynamic model increases slowly with the increase in the ratio of data and significantly outperforms the mix model. It suggests that the dramatic robustness of the dynamic model. As well, it can be anticipated that the prediction performance of the mix model can be improved when to learn with more data.

#### Statistical test

We perform a paired comparison *t*-test to verify if the performance improvement of the proposed approach over benchmark models is statistically significant. The paired sample *t*-test is a statistical procedure used to determine whether the mean difference between the two sets of observations is zero. Shifting to our problem, MACE of all patient samples was predicted using both our proposed models and baseline models, resulting in pairs of observations between each pair of models.

The performance of our approach showed considerable improvements regarding AUC in predicting MACE of ACS patients and the *t*-test showed in Table 3 demonstrated that there are indeed statistically significant differences between our model and benchmark models. As can be seen in Table 3, each model we implemented has a substantial difference compared to the others. All model pairs have a *p*-value< 0.05, which suggests that the proposed deep learning approach, especially the dynamic model, obtains a competitive and statistically significant performance in MACE prediction in comparison with the benchmark models.

**Table 3:** Statistical AUC differences in MACE prediction between models

Model	Boosted-RMTM	LR	Dynamic	Mix
Boosted-RMTM	/	5.98E-5**	5.46E-11**	0.018*
LR	/	/	4.68E-7**	1.17E-3**
Dynamic	/	/	/	1.16E-9**
Mix	/	/	/	/

**: *p*-value < 0.01; *: *p*-value < 0.05

## Discussions

With these experimental results, we summarize several interesting findings as follows:

- In most cases, our proposed dynamic model outperforms benchmark models for predicting MACE after ACS. The *p*-values between the proposed model and benchmark models show that there is a significant difference between the performances obtained by the employed models. Our proposed dynamic model has an average AUC of 0.713 and is thus the best MACE predictor. These findings confirm our assumption that leveraging dynamic treatment information contained in a large volume of heterogeneous EHR appears to boost the performance of MACE prediction, and has significant potential to meet the demand clinical prediction of other diseases, from a large volume of EHR in an open-ended fashion.
- With the gradual inclusion of more treatment information into learning for individuals, the prediction performance dramatically increases. The tendency of the curve in Fig. 5 arises as the hospitalization day per patient increases. As well, it is clearly to see that the curve surpasses 0.7 in terms of AUC after the number of hospitalization days is larger than five. It indicates that we need at least 5 days’ treatment information per patient to obtain the stable prediction results.
- It is not surprising to see from Fig. 6 that with sufficient training data samples, the proposed model can achieve a better prediction performance since deep learning method can achieve accurate representations from the big data. Due to the large amount of EHR data generated over time, we plan to investigate the suitability of deep neural networks for discovering nontrivial knowledge that best describe the inpatient treatment journeys and then improve the performance of MACE prediction.

Overall, compared with benchmark models, our model improves the performance of MACE prediction of ACS. Theoretically, many state-of-the-art machine learning algorithms, such as logistic regression, rely on aggregate features to produce a MACE prediction model based on static patient features and are not suitable for coping with the dynamic nature of treatment information during the hospitalization of ACS patients [35]. As a result, they lack the ability to model time-dependent co-variates in the observation window such that an individual’s progression mediated by dynamic treatment information cannot be reliably measured to improve the performance of predictive models in a continuous manner. To address this challenge, we utilize deep learning tacit to generate latent representations of dynamic treatment information for ACS patients from their heterogeneous EHRs. It provides a possible avenue to predict MACE in a real-time manner.

The experimental results were evaluated by clinical experts from Chinese PLA General hospital. In general, physicians from the hospital are satisfied with the prediction performance, and they indicate that the proposed model can provide a continuous MACE prediction service to monitor treatment processes of ACS patients predictively. It is also applicable to clinical decision support systems that recommend proper treatment interventions for physicians, which can significantly minimize the possibility of MACE occurrences.

### Limitations

Although our study reveals that the proposed model is useful in predicting MACE after ACS, there are complex and critical tasks that need to be further considered.

- For one thing, the dynamic nature of patient status is often essential/critical to the selection of treatment interventions. To address this challenge, we expect that our proposed model can incorporate richer execution information, e.g., vital signs, symptoms, and clinical observations on patient status, etc., into learning, which would make our proposed model more intelligent in the treatment adoption and MACE prevention.
- For the other thing, our proposed model neglects the causal relations between treatment interventions and effects. Note that causal effect analysis is useful to find out unexpected changes in treatment interventions and explain why scheduled treatment plans are changed to obtain the optimal treatment effects. As an open medical problem, the causal effect analysis can be benefited in mining a large scale of EHR data in a maximum-informative manner.

## Conclusion

This paper proposes a novel deep learning based approach to address the MACE prediction problem for ACS patients during their hospitalization. In comparison with existing ACS risk scoring models that can only rely on a small set of patient features, our proposed model can predict the occurrence probabilities well of MACE by utilizing a large volume of longitudinal and heterogeneous EHR data, especially the dynamic treatment information. The proposed model relies on a Bi-LSTM-based deep learning structure to aggregate dynamic treatment information in patients’ hospitalization. Then, the extracted latent dynamic treatment features are concatenated with static patient features to induce a regression layer for MACE prediction. Experiments conducted on a real clinical dataset illustrate that our proposed model can reach a highly competitive performance in predicting MACE for ACS patients, compared to state-of-the-art machine learning models, e.g., logistic regression, and the boosted-RMTM model proposed in our previous work.

We plan to carry out our future work along two directions. First, we intend to conduct a large scale of experiments and evaluate the performance of our proposed model on a larger scale of EHRs with more complex diseases. In addition, we plan to develop and deploy a dynamic MACE prediction service in treatment processes of ACS patients. As advocated by our clinical collaborators, the dynamic MACE prediction service can support healthcare professionals for estimating clinical risks of ACS patients nearly real-time and therefore adjusting/scheduling appropriate treatment interventions to reduce the occurrences of MACE in a continuous and predictive manner.

## Abbreviations

ACC: Accuracy
ACS: Acute coronary syndrome
AUC: Area under the curve
Bi-LSTM: Bidirectional long short term memory
EHR: Electronic health record
LR: Logistic regression
MACE: Major adverse cardiac event
RNN: Recurrent neural network
